# Supplementary material for: Enhancing rice growth and yield with weed endophytic bacteria Alcaligenes faecalis and Metabacillus indicus under reduced chemical fertilization
Source: PLoS One. 2024 May 16;19(5):e0296547. doi: 10.1371/journal.pone.0296547 (PMC11098348; doi:10.1371/journal.pone.0296547)
Supplement: S1 File — (DOCX) [file pone.0296547.s001.docx]

Supplementary Information

Title: Enhancing Rice Growth and Yield with Weed Endophytic Bacteria *Alcaligenes faecalis* and *Metabacillus indicus* Under Reduced Chemical Fertilization

**Authors:** Kaniz Fatema^1^, Nur Uddin Mahmud^1^, Dipali Rani Gupta^1^, Md Nurealam Siddiqui^2^, Tahsin Islam Sakif^3^, Aniruddha Sarker^4^, Andrew G Sharpe^5^, Tofazzal Islam^1*^

^1^Institute of Biotechnology and Genetic Engineering (IBGE), Bangabandhu Sheikh Mujibur Rahman Agricultural University, Gazipur 1706, Bangladesh

^2^Department of Biochemistry and Molecular Biology, Bangabandhu Sheikh Mujibur Rahman Agricultural University, Gazipur 1706, Bangladesh

^3^Lane Department of Computer Science and Electrical Engineering, West Virginia University, Morgantown, WV 26506-6109, USA

^4^Residual Chemical Assessment Division, National Institute of Agricultural Sciences, Rural Development Administration, Jeollabuk-do 55365, Republic of Korea

^5^Global Institute for Food Security, University of Saskatchewan, Saskatoon, SK, Canada

This Supplementary file contains the following information.

**Supplementary Figure S1** Weather status during the pot experiment at Banga Bandhu Sheikh Mujibur Rahman Agricultural University (BSMRAU), Salna, Gazipur-1701, Bangladesh.

**Supplementary Figure S2** Image of isolated bacterial strains from various major rice weeds.

**Supplementary Figure S3** Effects of various bacterial isolates on the rice seedlings at DAI 15 germination rate after treated with different strains.

**Supplementary Figure S4** Characterization for plant growth promoting traits of the isolated bacteria.

**Supplementary Figure S5** Neighbor-joining phylogenetic tree based on 16S rRNA gene sequence.

**Supplementary Table S1** Primer used for molecular identification of bacterial isolates.

**Supplementary Table 2** Colony characteristics of isolated endophytic bacteria from various rice associated weeds collected from field laboratory of BSMRAU.


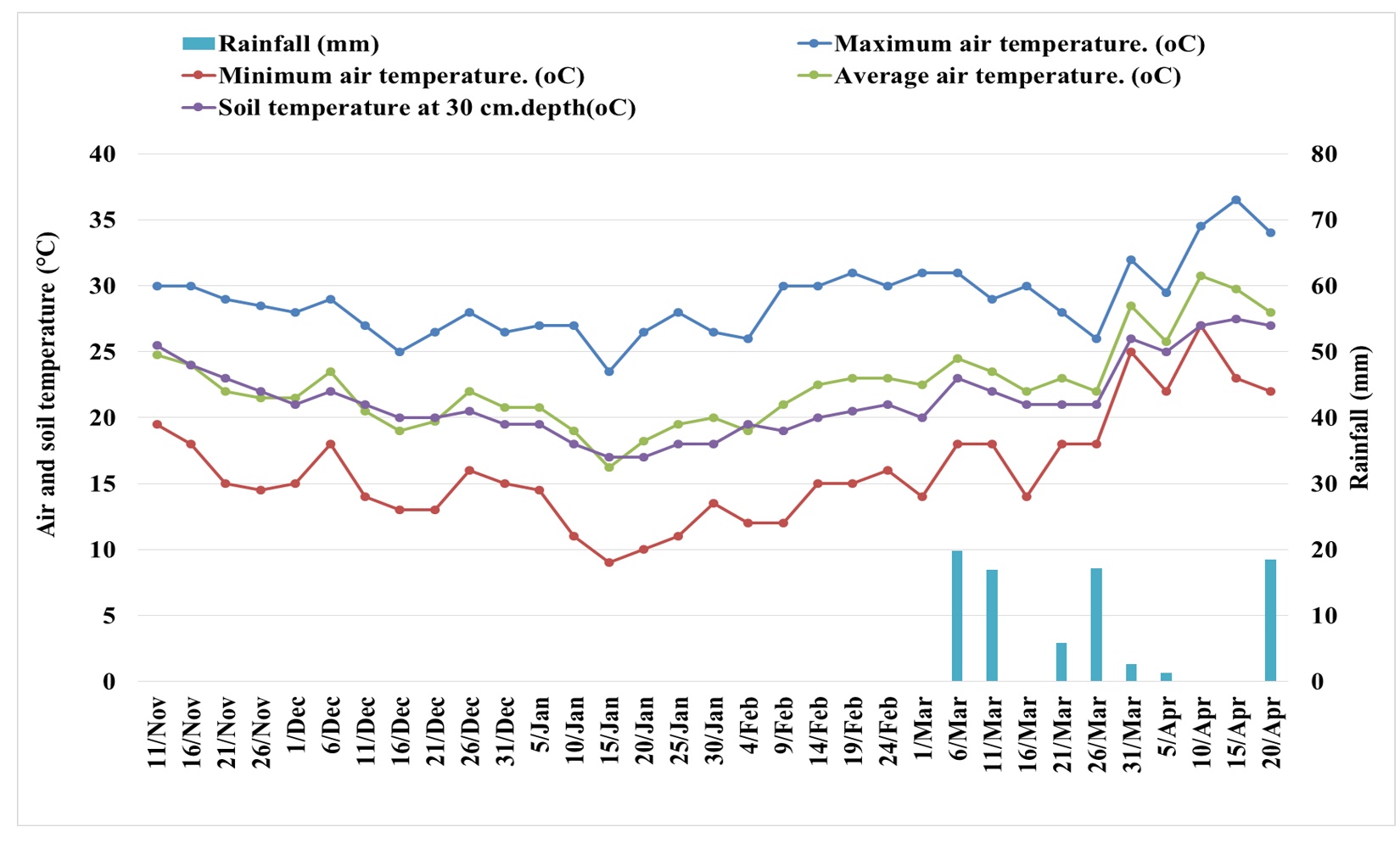


**Supplementary Figure S1** Weather status during the pot experiment at Banga Bandhu Sheikh Mujibur Rahman Agricultural University (BSMRAU), Salna, Gazipur-1701, Bangladesh.


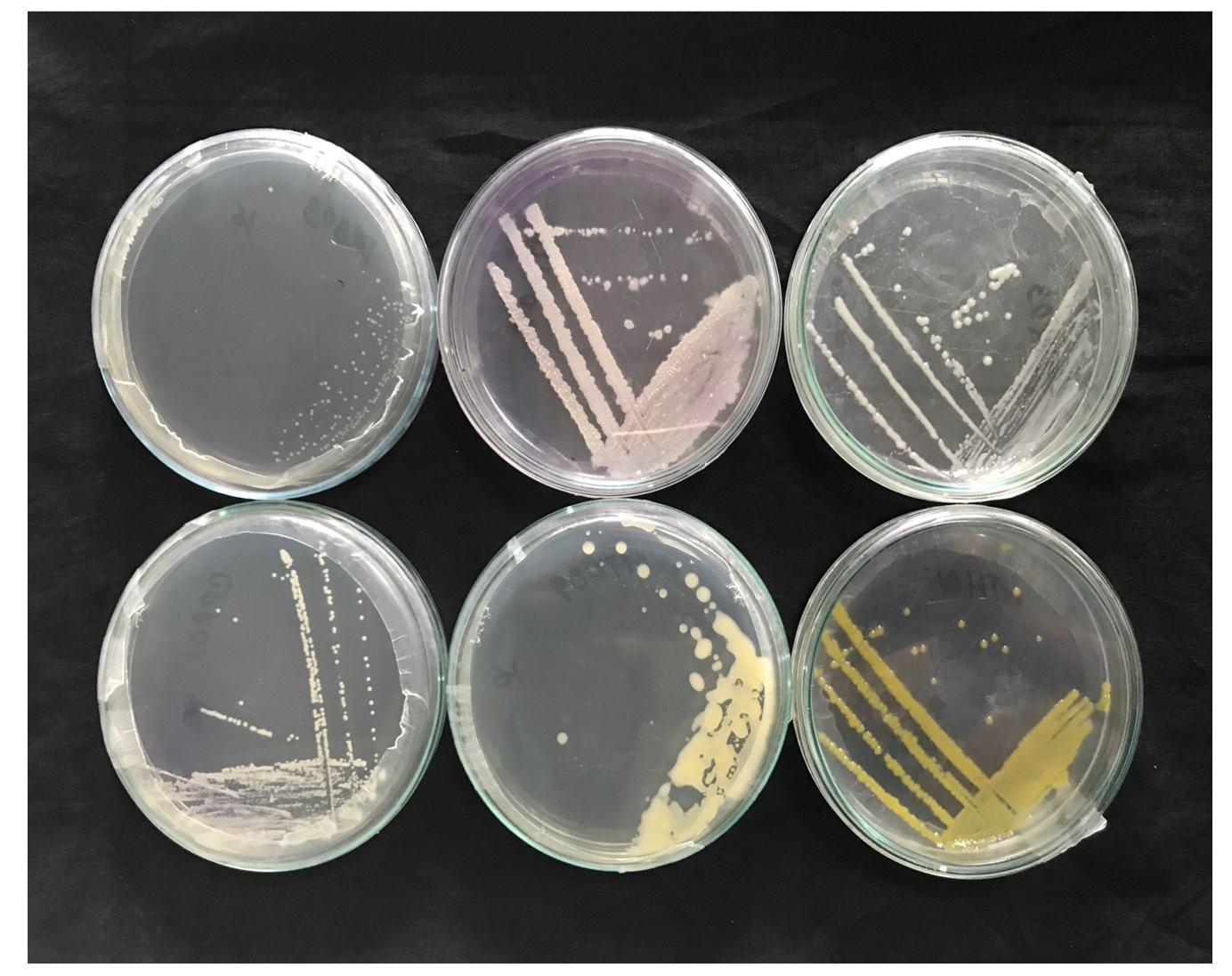


**Supplementary Figure S2** Image of isolated bacterial strains from various major rice weeds.


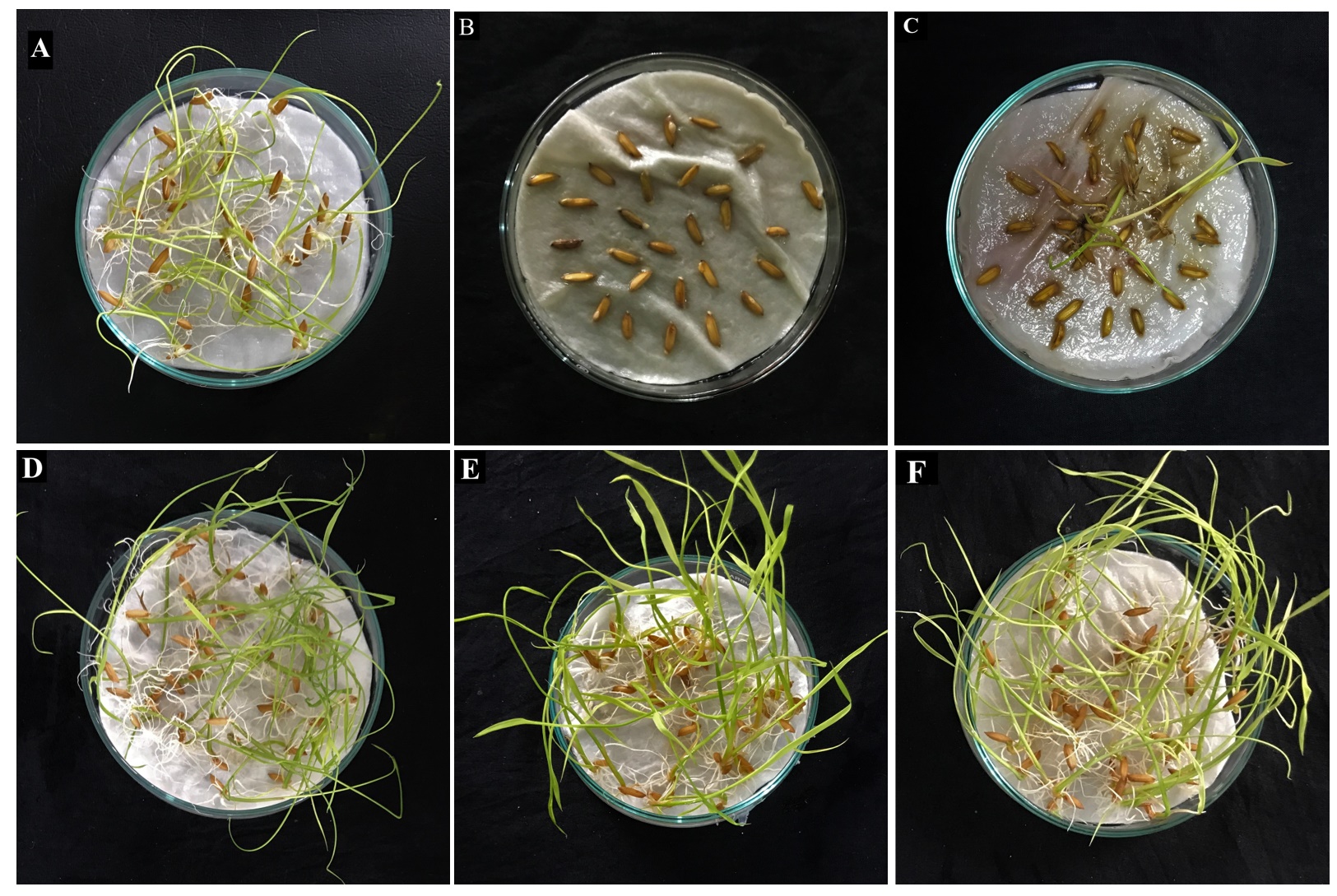


**Supplementary Figure S3** Effects of various bacterial isolates on the rice seedlings at DAI 15 germination rate after treated with different strains (A) untreated seeds (control), (B) seeds treated with BTCS01, (C) seeds treated with BTBS01, (D) seeds treated with BTMT07, (E) seed treated with BTCP01, (F) seeds treated with BTDR03.


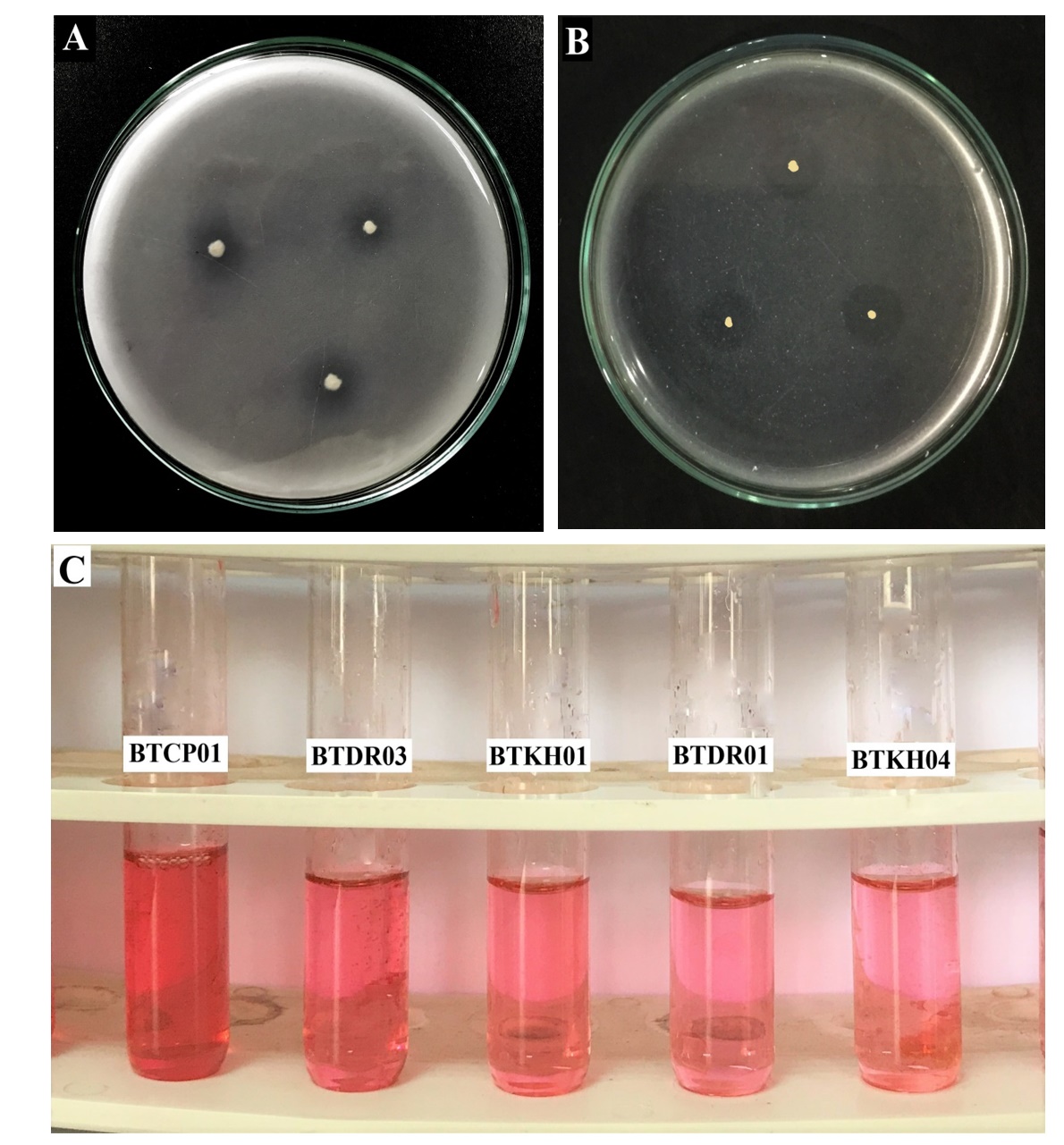


**Supplementary Figure S4** Characterization for plant growth promoting traits of the isolated bacteria, (A) BTCP01 exhibited halozone silubilizing inorganic phosphate on NBRIP agar medium, (B) BTDR03 exhibited halozone solibilizing mineral potassium on modified Aleksandrov media, (C) BTCP01 and BTDR03 produced Indol Acetic Acid.


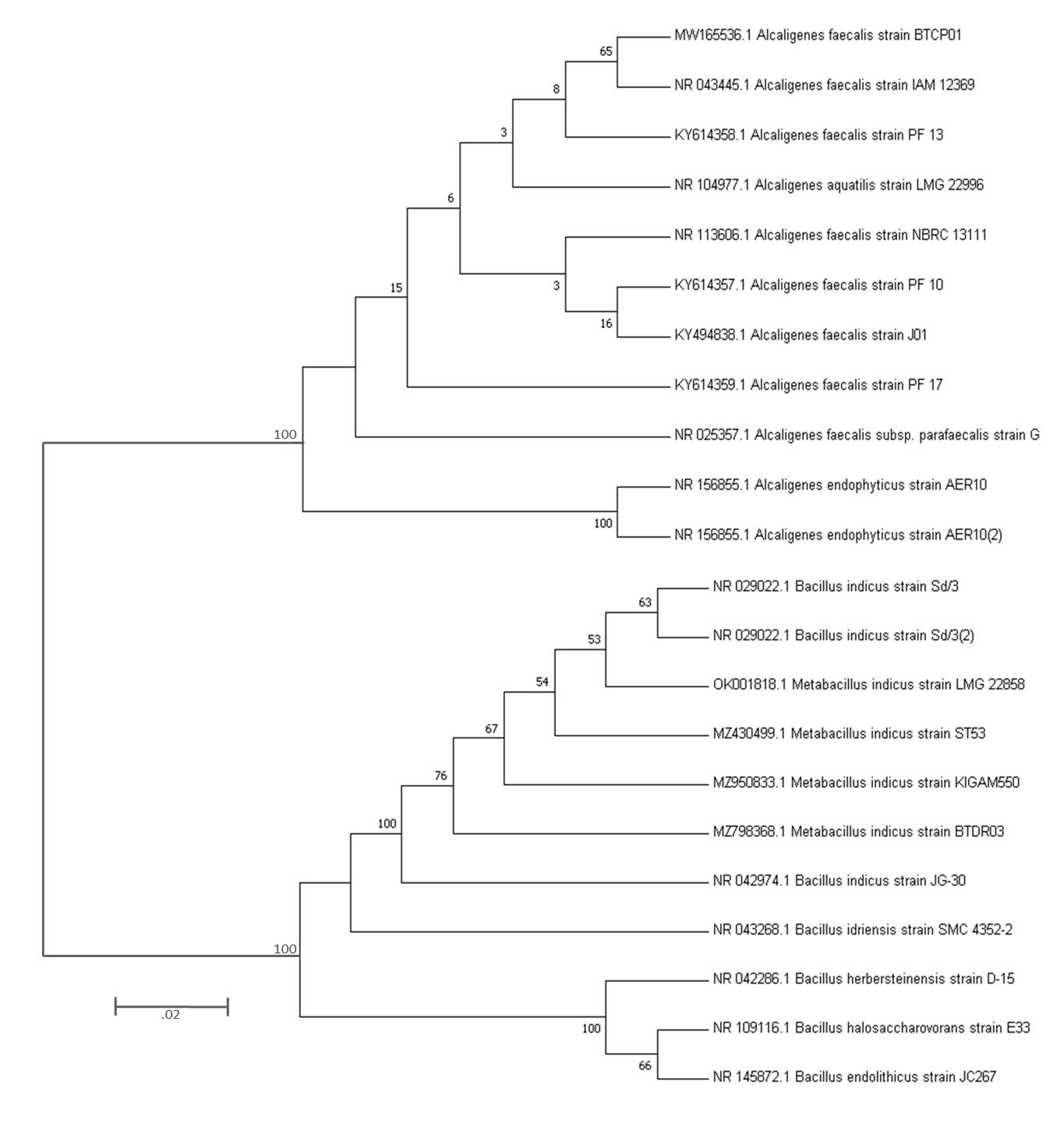


**Supplementary Figure S5** Neighbor-joining phylogenetic tree based on 16S rRNA gene sequence.

The significance of each branch is indicated by a bootstrap value based on 1000 replications.

**Supplementary Table S1** Primer used for molecular identification of bacterial isolates.

| Primer | Primer sequence | Thermal Cycle | Amplicon size (bp) |
| --- | --- | --- | --- |
| *Nif H* | F- GTTTTACGGCAAGGGCGGTATCGGCA  R- TCCTCCAGCTCTCCATGGTGATCsG | 94-5m, 94-1m, 50-1m, 72-2m, 72-10m, 4-∞; 35 cycle | 460 |
| *ipdC* | F- GAAGGATCCCTGTTATGCGAACC  R- CTGGGGATCCGACAAGTAATCAGGC | 95-5m, 95-30s, 55-30s, 72-1m, 72-5m, 4-∞; 35 cycle | 1700 |
| pqqC | F - CAGGGCTGGGTCGCCAACC  R- CATGGCATCGAGCATGCTCC | 96-10m, 96-30s, 54-30s, 72-1m, 72-10m, 4-∞; 30 cycle | 546 |
| kdpA | F- GAT TGA ACG GCC TAC TGG TC  R- CTG GAT CTT CTT GCC GAG AT | 95-10m, 95-30s, 51-30s, 72-30s, 72-10m, 4-∞; 35 cycle | 950 |

**Supplementary Table 2** Colony characteristics of isolated endophytic bacteria from various rice associated weeds collected from field laboratory of BSMRAU.

| SN | Strain name | Host | Plant parts | Colony color and morphology | | Seedling assay | | | | | | IAA production  ( μg/mL) |
| --- | --- | --- | --- | --- | --- | --- | --- | --- | --- | --- | --- | --- |
|  |  |  |  |  |  | Germination percentage (%) | Germination percentage higher than control (%) | Shoot length  (cm) | Root length  (cm) | Shoot fresh  weight  (gm) | Root fresh weight  (gm) |  |
|  |  |  |  | Colony color | Colony Shape |  |  |  |  |  |  |  |
| 1 | BTCP01 | *Eleusine indica* | Shoot | White | Irregular | 96 | 22.04 | 10.15 ±0.028c | 5.10 ± 0.057bc | 0.98 ±0.003a | 0.94 ±0.003a | 42.51 |
| 2 | BTCP02 | *E. indica* | Shoot | White | Irregular | 0 | 0 | 0 | 0 | 0 | 0 | 0 |
| 3 | BTCP03 | *E. indica* | Shoot | Orange | Circular | 92 | 16.96 | 9.25 ±0.028e | 3.75 ±0.028gh | 0.82 ±0.005c | 0.64±0.003b | 38.63 |
| 4 | BTCP04 | *E. indica* | Shoot | White | Circular | 88 | 11.87 | 7.35 ±0.104h | 2.75 ±0.028m | 0.49 ±0.003k | 0.33 ±0.005hi | 44.54 |
| 5 | BTCP05 | *E. indica* | Shoot | White | Circular | 66 | 0 | 4.95 ±0.028k | 1.35 ±0.028n | 0.37 ±0.005m | 0.21 ±0.003kl | 0 |
| 6 | BTUL01 | *Imperata cylindrica* | Shoot | White | Irregular | 0 | 0 | 0 | 0 | 0 | 0 | 0 |
| 7 | BTUL02 | *I.cylindrica* | Shoot | White | Circular | 0 | 0 | 0 | 0 | 0 | 0 | 0 |
| 8 | BTDR01 | *Cynodon dactylon* | Shoot | White | Circular | 92 | 16.96 | 7.50 ±0.057h | 6.25 ±0.028a | 0.73 ±0.003e | 0.50 ±0.003e | 36.69 |
| 9 | BTDR02 | *C. dactylon* | Shoot | White | Circular | 88 | 11.87 | 7.50 ±0.057h | 4.00 ± 0f | 0.62 ±0.003h | 0.45 ±0.003f | 40.12 |
| 10 | BTDR03 | *C. dactylon* | Shoot | Yellow | Irregular | 92 | 16.96 | 10.30 ±.050b | 5 ± 0.028cd | 0.79 ±0.005d | 0.58 ±0.005c | 40.86 |
| 11 | BTDR04 | *C. dactylon* | Shoot | White | Circular | 76 | 0 | 6.75 ±0.028i | 2.85 ±0.028lm | 0.66 ±0.005g | 0.35 ±0.005h | 18.24 |
| 12 | BTDR05 | *C. dactylon* | Shoot | White | Circular | 72 | 0 | 6.75 ±0.028i | 1.6 ± 0.057m | 0.36 ±0.005m | 0.19 ±0.003l | 22.98 |
| 13 | BTAG01 | *Digitaria sanguinalis* | Root | Ivory | Circular | 0 | 0 | 0 | 0 | 0 | 0 | 0 |
| 14 | BTAG02 | *D. sanguinalis* | Root | Ivory | Circular | 0 | 0 | 0 | 0 | 0 | 0 | 0 |
| 15 | BTAG03 | *D. sanguinalis* | Root | yellow | Circular | 40 | 0 | 0 | 0 | 0 | 0 | 0 |
| 16 | BTAG04 | *D. sanguinalis* | Root | White | Irregular | 0 | 0 | 0 | 0 | 0 | 0 | 0 |
| 17 | BTCS01 | *Echinochloa colonum* | Shoot | White | Circular | 0 | 0 | 0 | 0 | 0 | 0 | 0 |
| 18 | BTCS02 | *E. colonum* | Shoot | Orange | Circular | 0 | 0 | 0 | 0 | 0 | 0 | 0 |
| 19 | BTCS03 | *E. colonum* | Shoot | White | Circular | 0 | 0 | 0 | 0 | 0 | 0 | 0 |
| 20 | BTCS04 | *E. colonum* | Root | White | Circular | 0 | 0 | 0 | 0 | 0 | 0 | 0 |
| 21 | BTKH01 | *Saccharum spontaneum* | Root | White | Circular | 84 | 6.79 | 4.20 ±0.057l | 0.95 ± 0.028p | 0.43 ±0.003l | 0.31 ±0.003i | 37.94 |
| 22 | BTKH02 | *S. spontaneum* | Root | White | Circular | 88 | 11.87 | 9.00 ±0.020f | 4.25 ± 0.028e | 0.83 ±0.003c | 0.66 ±0.003b | 34.49 |
| 23 | BTKH03 | *S. spontaneum* | Root | Brown | Circular | 96 | 22.04 | 4.45 ±0.028l | 3.25 ± 0.028e | 0.34 ±0.003m | 0.26 ±0.005j | 37.65 |
| 24 | BTKH04 | *S. spontaneum* | Root | White | Circular | 96 | 22.04 | 6.75 ±0.028i | 5.25 ±0.028b | 0.53 ±0.005j | 0.26 ±0.005j | 36.2 |
| 25 | BTKH05 | *S. spontaneum* | Root | Yellow | Circular | 0 | 0 | 0 | 0 | 0 | 0 | 0 |
| 26 | BTKH06 | *S. spontaneum* | Root | White | Irregular | 88 | 11.87 | 4.25 ±0.0401 | 1.15 ±0.028o | 0.48 ±0.003k | 0.21 ±0.003kl | 33.50 |
| 27 | BTKH07 | *S. spontaneum* | Root | Ivory | Circular | 68 | 0 | 7.50±0.057h | 4.25 ±0.028e | 0.55 ±0.005ij | 0.55 ±0.005d | 16.20 |
| 28 | BTAR01 | *Leersia hexanda* Sw. | Shoot | White | Irregular | 40 | 0 | 0 | 0 | 0 | 0 | 0 |
| 29 | BTAR02 | *L. hexanda* Sw. | Shoot | Yellow | Circular | 0 | 0 | 0 | 0 | 0 | 0 | 0 |
| 30 | BTAR03 | *L. hexanda* Sw. | Shoot | Ivory | Irregular | 0 | 0 | 0 | 0 | 0 | 0 | 0 |
| 31 | BTBS01 | *Echinochloa crussgalli* | Shoot | Yellow | Circular | 32 | 0 | 0 | 0 | 0 | 0 | 0 |
| 32 | BTBS02 | *E. crussgalli* | Shoot | White | Circular | 0 | 0 | 0 | 0 | 0 | 0 | 0 |
| 33 | BTBS03 | *E. crussgalli* | Root | White | Irregular | 48 | 0 | 0 | 0 | 0 | 0 | 0 |
| 34 | BTBS04 | *E. crussgalli* | Root | White | Circular | 0 | 0 | 0 | 0 | 0 | 0 | 0 |
| 35 | BTBS05 | *E. crussgalli* | Root | White | Circular | 36 | 0 | 0 | 0 | 0 | 0 | 0 |
| 36 | BTKP01 | *Dactyloctenium aegyptium* | Root | White | Circular | 0 | 0 | 0 | 0 | 0 | 0 | 0 |
| 37 | BTKP02 | *D. aegyptium* | Root | Yellow | Circular | 36 | 0 | 0 | 0 | 0 | 0 | 0 |
| 38 | BTKP03 | *D. aegyptium* | Root | Ivory | Circular | 0 | 0 | 0 | 0 | 0 | 0 | 0 |
| 39 | BTMT01 | *Cyperus rotundus* | Shoot | White | Circular | 92 | 16.96 | 11.25 ±0.028a | 3.85 ±0.028fg | 0.86 ±0.003b | 0.53 ±0.005d | 36.88 |
| 40 | BTMT02 | *C. rotundus* | Shoot | White | Circular | 88 | 11.87 | 8.25 ±0.028g | 3.00 ±0.00jk | 0.43 ±0.003l | 0.23±0.003k | 42.78 |
| 41 | BTMT03 | *C. rotundus* | Shoot | White | Circular | 92 | 16.96 | 7.50 ±0.028h | 5.25 ±0.028b | 0.56 ±0.003i | 0.31 ±0.005c | 38.90 |
| 42 | BTMT04 | *C. rotundus* | Root | Yellow | Circular | 0 | 0 | 0 | 0 | 0 | 0 | 0 |
| 43 | BTMT05 | *C. rotundus* | Root | Orange | Circular | 67.58 | 0 | 10.30 ±0.057b | 4.35 ±0.028e | 0.71 ±0.005f | 0.42 ±0.005g | 28.20 |
| 44 | BTMT06 | *C. rotundus* | Root | White | Circular | 94.66 | 20.34 | 10.60 ±0.057b | 4.40± 0.057e | 0.77 ±0.005d | 0.43 ±0.003fg | 35.20 |
| 45 | BTMT07 | *C. rotundus* | Root | Yellow | Irregular | 88 | 11.87 | 9.80 ±0.057d | 3.10 ± 0.057ij | 0.66 ±0.005g | 0.45 ±0.005f | 34.47 |
| 46 | Control |  | 0 | 0 | 0 | 78.66 | 22.04 | 6.20 ±0.028j | 4.25 ± 0.057d | 0.54 ±0.005j | 0.32 ±0.0053i |  |
