# Supplementary material for: Enhancing rice growth and yield with weed endophytic bacteria Alcaligenes faecalis and Metabacillus indicus under reduced chemical fertilization
Source: PLoS One. 2024 May 16;19(5):e0296547. doi: 10.1371/journal.pone.0296547 (PMC11098348; doi:10.1371/journal.pone.0296547)
Supplement: Attachment — Submitted filename: Response to reviewer comments_R2.docx [file pone.0296547.s003.docx]

**Responses to the Reviewer’s Comments**

**Title of the Manuscript:** Enhancing Rice Growth and Yield with Weed Endophytic Bacteria Alcaligenes faecalis and Metabacillus indicus Under Reduced Chemical Fertilization

**Manuscript No.** PONE-D-23-42245R1

**Dear Editor,**

We are deeply grateful for the opportunity to revise our manuscript for your esteemed journal. We extend our sincere appreciation to you and the diligent reviewers for their invaluable feedback, which has significantly enhanced the quality of our work. We have meticulously addressed each comment and incorporated necessary revisions to improve the clarity and precision of our manuscript.

Please find below a detailed point-by-point response to the reviewers' comments:

**Review Comments to the Author**

Reviewer #1: The study investigating the potential of endophytic bacteria from native weeds as biofertilizers presents valuable insights into sustainable agricultural practices. The manuscript is well-written and addresses an important gap in the literature regarding the plant growth-promoting abilities of weed endophytes. However, to strengthen the impact and clarity of the findings, few minor modifications are recommended:

**Our response**: Many thanks for the encouraging comments and valuable comments for improvement of the impact and clarity of the findings.

1. Clarification of Methodology: Provide a more detailed description of the isolation and identification methods used for endophytic bacteria from native weeds. This will enhance reproducibility and aid readers in understanding the experimental procedures.

**Our response:** Thank you. We have provided a detailed description of the isolation process for endophytic bacteria from native weeds, including specific procedures, media composition, and isolation conditions under the headline ***2.2. Collection of plant materials and isolation of bacteria.***

2. Taxonomic Identification: While the tentative identification of the two promising strains (BTCP01 and BTDR03) based on 16S rRNA gene phylogeny is informative, consider additional validation methods, such as whole-genome sequencing or multilocus sequence analysis, to confirm their taxonomic classification.

**Our response:** We value the reviewer's insightful suggestion. While whole-genome sequencing offers more definitive taxonomic classification, its implementation involves considerable time, complexity in data analysis, and costs. Regrettably, these constraints preclude its inclusion within the timeframe allocated for revising this manuscript. Given that 16S rRNA gene sequencing is generally acceptable for preliminary identification of environmental bacteria, we will explore the recommended whole-genome sequencing and data annotation in our future projects.

3. Quantitative Data Presentation: Present quantitative data on mineral phosphate and potash solubilization activities, as well as IAA production, in a clear and organized manner. Tables or graphs summarizing these results would facilitate interpretation and comparison between isolates.

**Our response:** We have included detailed tables and figures in Supplementary Table 2, summarizing the mineral phosphate and potash solubilization activities of the isolated strains. Statistical analyses were conducted where applicable, and significance levels were specified.

4. Gene Detection Analysis: Provide more details on the methodology and significance of detecting the nifH and ipdC genes in the genomes of the selected isolates. Discuss the potential role of these genes in plant-microbe interactions and their implications for biofertilizer applications.

**Our response:** Thank you for this important suggestion. We have provided the details on ***2.6. Design of primers for amplification of nifH and ipdC genes****.*

5. Agronomic Performance Assessment: Expand on the methods used to evaluate the agronomic performance of rice variety BRRI dhan29 following inoculation with endophytic bacterial strains. Include parameters such as plant height, root morphology, and nutrient uptake efficiency to comprehensively assess the plant growth-promoting effects.

**Our response:** This is indeed a good suggestion. However, in this project, we did not evaluate the root morphology and nutrient uptake efficiency. This suggestion will be helpful in our next project.

6. Statistical Analysis and Interpretation: Enhance the statistical analysis by including appropriate tests to determine the significance of differences observed in agronomic parameters between treatment groups and control. Provide a detailed interpretation of the results, discussing the practical implications for sustainable rice production.

**Our response:** Thank you so much for this suggestion. Our main target was to identify potential bacteria which can improve the growth and yield with various fertilizer doses. And we have provided detailed interpretation under the headline ***3.4. Promotion of growth and yield of rice cv. CV. BRRI dhan29***.

We hope that our revised version will be accepted by the reviewers and you. Should you have any further queries and suggestions, please feel free to contact me.

Thank you once again for your time and consideration of our manuscript.

**Best regards,**

On behalf of the authors,

Tofazzal Islam	March 19, 2024

..........................................

**Md Tofazzal Islam, Ph D**

*Fellow of the American Phytopathological Society (APS), Bangladesh Academy of Sciences (BAS), The World Academy of Sciences (TWAS), and Bangladesh Academy of Agriculture (BAAG)*

*&*

Professor and Founding Director, Institute of Biotechnology and Genetic Engineering (IBGE),

Bangabandhu Sheikh Mujibur Rahman Agricultural University, Gazipur-1706
BANGLADESH
Tel. +88-02-9205310-14 Extn. 2252
Fax: +88-02-9205333

Cell: +88-0171-4001414

***Editor: Physiologia Plantarum***

***Associate Editor: Frontiers in Microbiology***

***Editor: Scientific Reports***

***Academic Editor: PLOS ONE***

<https://ibge.bsmrau.edu.bd/tofazzal/>

<http://www.researchgate.net/profile/Md_Tofazzal_Islam>

<http://orcid.org/0000-0002-7613-0261>

<https://www.apsnet.org/members/give-awards/awards/2022Awardees/Pages/Fellow_Islam_Tofazzal.aspx>

[https://twas.org/directory/islam-md-tofazzal](https://twas.org/directory/islam-md-tofazzal?fbclid=IwAR2ad8KtHqYO7loDcy8pGX1-2IOzjusk8iinZKZJhVqK441Tc5qR9lYaYLI)
