# Supplementary material for: Enhancing rice growth and yield with weed endophytic bacteria Alcaligenes faecalis and Metabacillus indicus under reduced chemical fertilization
Source: PLoS One. 2024 May 16;19(5):e0296547. doi: 10.1371/journal.pone.0296547 (PMC11098348; doi:10.1371/journal.pone.0296547)
Supplement: Attachment — Submitted filename: Response to reviewer comments.docx [file pone.0296547.s002.docx]

Dated: 9 February 2024

Editorial Office

PLOS ONE

*Subject: Revision of manuscript # PONE-D-23-42245*

**Dear Dr Sofia Isabel Almeida Pereira,**

First of all, we gratefully appreciate your time and interest on handling our manuscript. We have revised the above-mentioned manuscript according to the suggestions of the two reviewers. A point-wise reply to the reviewer´s comments is presented at the end of this letter. We edited the manuscript text and re-arranged the Figure in order to provide an improved version that will hopefully meet the standards for publication at *PLOS ONE*. All alterations were made highlighting by the red color in Word, and are therefore properly highlighted in the revised manuscript.

All listed co-authors have approved the resubmission of this manuscript. The authors declare no conflict of interest.

We gratefully appreciate your time and interest. We look forward to hearing from you.

Sincerely,

Tofazzal Islam

*Fellow of the American Phytopathological Society (APS), Bangladesh Academy of Sciences (BAS), The World Academy of Sciences (TWAS), and Bangladesh Academy of Agriculture (BAAG)*

*&*

Professor and Founding Director, Institute of Biotechnology and Genetic Engineering (IBGE),

Bangabandhu Sheikh Mujibur Rahman Agricultural University, Gazipur-1706
BANGLADESH
Tel. +88-02-9205310-14 Extn. 2252
Fax: +88-02-9205333

Cell: +88-0171-4001414

***Editor: Physiologia Plantarum***

***Associate Editor: Frontiers in Microbiology***

***Editor: Scientific Reports***

***Academic Editor: PLOS ONE***

<https://ibge.bsmrau.edu.bd/tofazzal/>

**Our Response to reviewer comments**

**Reviewer #1:**

1. Clarity of Experimental Design:
- The experimental design and methodology need further clarification. Provide a detailed description of the isolation process for endophytic bacteria from native weeds, including specific procedures, media composition, and isolation conditions. Additionally, elaborate on the criteria used for selecting the two promising strains (BTCP01 and BTDR03) and the rationale behind their identification.

**Response:** Thank you for your nice comments. We have provided a detailed description of the isolation process for endophytic bacteria from native weeds, including specific procedures, media composition, and isolation conditions under the headline ***2.2. Collection of plant materials and isolation of bacteria*** (Line 90-109). However, the criteria used for selecting the two promising strains (BTCP01 and BTDR03) and the rationale behind their identification were elaborated under the headline ***3.1. Isolation, biochemical and molecular characterization of weed endophytic bacteria*** (Line 241-257).

2. Genetic Characterization:
- Strengthen the genetic characterization section by providing more details on the detection and analysis of the nifH and ipdC genes in the genomes of BTCP01 and BTDR03. Include the primers used, amplification conditions, and the significance of these genes in the context of plant growth promotion.

**Response:** We have provided the detailed on the detection and analysis of *nifH* and *ipdC* genes in the genomes of BTCP01 and BTDR03 in Supplementary Table S1. The significance of these genes in the context of plant growth promotion were explained in Line 331-336.

3. Data Presentation:
- Revise the presentation of results to enhance clarity. Provide clear tables or figures summarizing the mineral phosphate and potash solubilization activities of the isolated strains. Include statistical analyses where applicable and specify the significance levels.

**Response:** Thank you. We have provided the detailed on clear tables and figures summarizing the mineral phosphate and potash solubilization activities of the isolated strains in Supplementary Table 2, where we have also included statistical analysis for identifying the significance levels by different lettering.

4. Root Dipping and Spraying Experiment:
- Elaborate on the methodology employed for the application of isolates BTCP01 and BTDR03 through root dipping and spraying at the flowering stage. Include details on the concentration, frequency, and duration of the applications.

**Response:** Thank you. We have provided the detailed in the Line number 215-226.

5. Agronomic Performance Assessment:
- Provide a more comprehensive analysis of the agronomic performance of rice variety BRRI dhan29. Include additional parameters such as plant height, tiller number, and any observed morphological changes. Consider incorporating statistical analyses to support the significance of the observed effects.

**Response:** We have provided the detail agronomic performances under the headline 3.4. Promotion of growth and yield of rice cv. BRRI dhan29 (Line280-314).

6. Comparison with Control:
- Clearly outline the control conditions in the experiment and provide a detailed comparison with the treated plants. Specify the outcomes in terms of growth parameters and grain yields.

**Response:** Thank you for your crucial comments. We have provided the detailed under the headline 3.4. Promotion of growth and yield of rice cv. BRRI dhan29 (Line 280-314).

7. Discussion on Sustainable Agriculture:
- Expand the discussion on the potential implications of using weed endophytic bacteria as biofertilizers for sustainable agriculture. Address the broader context of reducing chemical fertilizer dependency and its impact on environmental sustainability.

**Response:**

Thank you for raising this important issue. We have added the context of reducing fertilizer dependency using plant-growth promoting bacteria in introduction (Line 60-66). To avoid the redundancy, we did not discuss this issue again in discussion section.

8. Concluding Remarks:
- Strengthen the concluding remarks by summarizing the key findings and their implications for sustainable rice production. Discuss the significance of the observed increase in grain yields and its potential contribution to agricultural practices.

**Response:** We have now added a key finding in concluding remarks in Line (369-375).

9. Language and Style:
- Review and improve the clarity of language and style throughout the manuscript. Ensure consistency in terminology and provide definitions or explanations for any specialized terms. Proofread for grammatical accuracy and overall readability. Addressing these major modifications will enhance the clarity, rigor, and overall quality of the manuscript.

**Response:** Thank you. We have improved the clarity of language and style throughout the manuscript thus definitely enhanced the clarity, rigor, and overall quality of the manuscript.

#############################################################################

**Reviewer #2:**

-It would have been better to design the experiment using a treatment containing a mixture of the two isolates.

**Response:** Thank you for your valuable comments. For a clear understanding, we provided the isolate performances under different fertilizer levels in Figures 3 and 4.

-It was necessary to ensure that the surface sterilization process of plant samples was successful; otherwise, you cannot confirm that the isolated bacteria are endophytes.

**Response:** Thank you for your valuable comments. Yes, surface sterilization was successful, therefore we found significant differences in germination rate, shoot length, root length, shoot fresh weight, and root fresh weight (Supplementary Table S2).

-Lines 99, 100: you reported that “seeds of the rice variety CV. BRRI dhan29 were procured from the Bangladesh Rice Research Institute (BRRI) for use in pot experiments to isolate endophytic bacteria” indicating isolation from rice seeds, while you reported isolation from grasses. Explain?

**Response:** Sorry for this mistake. Now we have corrected the statement (Line 99-100).

-In the indole production experiment, why did you use that concentration of L-tryptophan (1 ml of 0.2%)?

**Response:** Thank you for your valuable comments. The production of **indole-3-acetic acid (IAA)**, a plant hormone, by bacteria using **L-tryptophan** as a precursor. According to some studies, the optimal conditions for IAA production vary depending on the bacterial strain and the culture medium. For example, Rhizobium sp. [produced the highest amount of IAA (90.21 μg/ml) in culture media supplemented with 0.2% L-tryptophan and at an initial pH of 9](https://jgeb.springeropen.com/articles/10.1186/s43141-020-00090-2).

-The results should include a summary of the seedling assay results, especially since you chose the best isolates according to the results of the germination rate.

**Response:** Thank you for your valuable comments. We have added a summary in Line 269-273.

- Line 107, 108, 238: If the nutrient medium contains agar, “nutrient broth agar medium” must be
Corrected to “nutrient agar medium”

**Response:** Thank you. We have corrected the error throughout the manuscript.

-Line 270: “CPR01 and DRB03”, these codes do not match any of the codes for the 45 isolates included in the study?

**Response:** Thank you. We have corrected the error.

- The results should be presented in a simple and direct way that highlights the important points of the manuscript
- The discussion must be improved
Typo errors
Line 33: write “Eleusine indica” in italic

**Response:** Thank you. We have corrected the error.

Line 96: correct “hexanda” to “hexandra”, correct “Burnyard” to “Barnyard”, correct “crussgalli” to “crus-galli”

**Response:** Thank you. We have corrected the error.

Line 184: delete “[PSI”

**Response:** Thank you. We have corrected the error.

Line 197: delete “[KSI”

**Response:** Thank you. We have corrected the error.

Line 623: correct rice variety name “BRRI dhan29”

**Response:** Thank you. We have corrected the error.

Line 329: correct “Nicotinia” to “Nicotiana”

**Response:** Thank you. We have corrected the error.

-The name of the rice variety is written as “BRRI dhan29 “, CV. BRRI dhan29” and sometimes as “cv. BRRI dhan29”, check it

**Response:** Thank you. We have corrected the error.

-Fig. 1, 2: letters (A-I) in the figure title corresponding to percentage of the chemical fertilizer does not agree with the meaning of the letters on the figures

**Response:** Thank you. We kept lettering for quick understanding and matching with figure legend.

-Fig. 3 B; delete the duplicated letter (B)
**Response:** Deleted.

Line 421: correct “nbacteria” to “bacteria”

**Response:** Thank you. We have corrected the error.

Lines 553, 559: write “Bacillus” in italic

**Response:** Thank you. We have corrected the error.

Line 575: write “Agrobacterium” in italic

**Response:** Thank you. We have corrected the error.

-Correct title of Fig. S3 “Effects of various bacterial isolates on the rice seedlings”

**Response:** Thank you. Corrected.

-The title written in the supplementary material is different from the title written in the main manuscript

**Response:** Thank you for pointing out this mistake. Now it has been corrected.

References
-References No. 5, 7, 8, 9, 34, 47, 55, 56, 61, 62, 63 and 64 are not present in the text.
-References cited in the text and missed from the list;
-Line 131: “Bergey et al. (1994).”
-Line 139: “Maniatis et al. (1982).”
-Line 143: “(Reysenbach et al., 1992)”
-Line 153: “(Saitou and Nei, 1987), (Kumar et al., 2016)”
-Line 168: Bric et al., 1991

**Response:** Reference list matching with text has corrected.

##############################################################################
